# Individual and Interactive Effects of Temperature and Watering Regime on Canola Growth and Physiological Characteristics

**DOI:** 10.1002/pei3.70030

**Published:** 2025-03-07

**Authors:** Emma D. McDormand, Mirwais M. Qaderi

**Affiliations:** ^1^ Department of Biology Mount Saint Vincent University Halifax Nova Scotia Canada

**Keywords:** *Brassica napus*, drought stress, fatty acid, heat stress, photosynthetic pigment, plant biomass

## Abstract

Although many studies have considered the effects of temperature and water on plants, the combined effects of these factors on canola ( *Brassica napus* ) growth, physiological traits, and fatty acids require more attention. Canola is an important oilseed crop in Canada and around the world and fatty acids act as regulators of stress signaling. We grew plants under two temperature regimes (22°C/18°C and 28°C/24°C; 16 h light and 8 h dark) and two watering regimes (well‐watered and water stressed) in controlled‐environment growth chamber for 3 weeks after 1 week of initial growth under 22°C/18°C. We measured growth, biomass, photosynthesis, fatty acids, and other physiological traits of plants. With respect to plant growth and physiological traits, as individual factor, higher temperatures decreased stem diameter, specific leaf mass, leaf water potential, and flavonoids, whereas water stress decreased stem height and diameter, leaf area and number, leaf mass, net CO_2_ assimilation, transpiration, and stomatal conductance, but increased leaf mass ratio (leaf dry mass/plant dry mass). In terms of interaction, higher temperatures increased plant biomass, chlorophyll (Chl) *a*, carotenoids, and total Chl in the well‐watered plants, but decreased all these traits in the water‐stressed plants. With respect to fatty acids as individual factor, higher temperatures decreased tricosanoic acid (C23:0), but increased heptadecanoic acid (C17:0). In terms of interaction, higher temperatures decreased *cis*‐10‐heptadecenoic acid (C17:1), elaidic acid (C18:1), arachidic acid (C20:0), and *cis*‐11,14‐eicosadienoic acid (C20:2) in the water‐stressed plants. Lower temperatures also decreased C17:1, C18:1, and C20:2 in the water‐stressed plants. Overall, palmitoleic acid (C16:1) was higher in the stem than in the leaf. This study revealed that higher temperatures combined with water stress decreased some physiological traits and fatty acids and, in turn, plant biomass. Further studies are required to determine the effects of multiple climate change components on fatty acids in canola and other oilseed crops.

## Abstract 2

This study revealed that higher temperatures combined with water stress decreased some physiological traits and fatty acids and, in turn, plant biomass.

## Graphical abstract

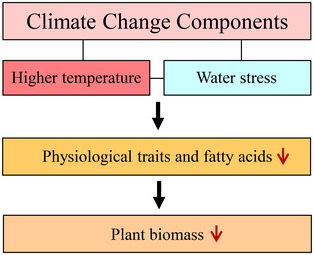

## 1 Introduction

Global climate change has been an ever‐increasing problem since the beginning of the Industrial Era (Porfirio et al. 2018). Anthropogenic activities have increased the levels of carbon dioxide (CO_2_) in the atmosphere. Along with methane (CH_4_), nitrous oxide (N_2_O), and water vapor, CO_2_ is a potent greenhouse gas that contributes to the “greenhouse effect” (Manning and Tiedemann 1995) and has been steadily increasing for the past 250 years (Masson‐Delmotte et al. 2021). Human activities have caused irreversible changes to the global climate that can have significant effects on crop production worldwide (Bita and Gerats 2013). Extreme conditions alter agricultural production, and concurrently affect the global market and food supply (Porfirio et al. 2018). With a reduction in crop yield and an expected population of 9 billion by the year 2050, global food insecurity may worsen. Global crop production must increase by 70% to accommodate the increased population (Bita and Gerats 2013). Heat stress will have the greatest effect on crop production, as drought conditions often accompany increased temperatures and can have detrimental effects on plant growth and yield (Bita and Gerats 2013; Nath et al. 2017; Qaderi, Martel, and Dixon 2019; Fan et al. 2024). It has been predicted that global temperatures will rise 1°C–4°C by 2100 (Masson‐Delmotte et al. 2021). The impacts of increased temperatures will vary across regional climates. For some species, increased temperatures in certain geographical locations may be beneficial, while in other species it can be detrimental (Qaderi and Reid 2009). Also, increased temperatures will alter precipitation patterns and cause more frequent and intense rainfall (Swann 2018).

Plants are affected differently by temperature throughout their life cycles; for example, crops during vegetative growth often have higher optimal temperatures than during reproductive development (Hatfield et al. 2011). Canola plants, grown under higher temperatures, have been shown to exhibit decreased stem height and diameter, leaf number and area, dry biomass, leaf area ratio (LAR), and water use efficiency (WUE) (Qaderi, Kurepin, and Reid 2006; Slauenwhite and Qaderi 2013; Reardon and Qaderi 2017). In addition, canola plants had increased leaf mass ratio (LMR), chlorophyll concentration, and specific leaf mass (SLM) (Qaderi, Kurepin, and Reid 2006). Higher temperatures have also been shown to enhance development, causing smaller plants, and shortening reproductive phase, resulting in reduced grain yield of crops. The largest impacts of increased temperature are seen during the reproductive stage, as it has a pronounced effect on pollen viability, and grain or fruit yield (Hatfield et al. 2011). It has been shown that increased temperature, as little as 1°C, decreased grain yield for economically important crops, such as rice ( *Oryza sativa* L.) by as much as 10% (Hatfield et al. 2011; Högy et al. 2013). A decrease in nutritional value has also been observed in barley (Högy et al. 2013). Heat stress has been shown to reduce overall biomass, fruit, and grain yield of crops (Barnabás, Jäger, and Fehér 2008; Bita and Gerats 2013; Slauenwhite and Qaderi 2013) through the inability of RuBisCO (ribulose‐1,5‐bisphosphate carboxylase/oxygenase) to fix carbon (Bita and Gerats 2013). Reduced activity of RuBisCO decreases photosynthesis and increases transpiration and stomatal conductance (Qaderi, Kurepin, and Reid 2006). The damage to chlorophyll and the photosynthetic apparatus is also associated with the production of reactive oxygen species (ROS) that can destroy proteins, lipids, and DNA (Barnabás, Jäger, and Fehér 2008; Bita and Gerats 2013) when produced in large quantities (Suzuki and Mittler 2006; Wilson et al. 2014). Moreover, increased temperatures alter the levels of phytohormones, such as salicylic acid, abscisic acid, and ethylene (Bita and Gerats 2013), and alter the expression of protective proteins, antioxidants, and heat shock proteins (Wahid et al. 2007; Barnabás, Jäger, and Fehér 2008; Bita and Gerats 2013).

Water scarcity has the potential to decrease crop yield and quality by altering plant growth and development and inducing detrimental physiological and biochemical processes (McDowell et al. 2008). During drought, plants often extend their root systems to reach water deep in the soil (Barnabás, Jäger, and Fehér 2008; Fang and Xiong 2015). Due to lack of resources in the shoots, plants under drought conditions often have decreased stem height and diameter, leaf number, and area, resulting in reduced biomass, as shown in canola (Qaderi, Kurepin, and Reid 2006). Reduced number and area of leaves lead to decreased photosynthetic rate. In the model plant *Arabidopsis thaliana* , it has been reported that plants may activate other drought stress responses, such as leaf blade curling to reduce surface area exposed to sunlight, thickening of the cuticular wax, and increased epidermal hairs to prevent dehydration (Fang and Xiong 2015).

Water deficits typically accompany elevated temperatures (Swann 2018). Many studies have looked at the effects of temperature (Wahid et al. 2007; Bita and Gerats 2013; Högy et al. 2013; Hatfield and Prueger 2015) and watering regime (Qaderi, Kurepin, and Reid 2006, 2012) on growth and physiological responses of canola. The effects of drought stress on fatty acid composition have been studied in the seeds of groundnuts (Dwivedi et al. 1996), soybeans (Dornbos and Mullen 1992), sunflowers (Petcu, Arsintescu, and Stanciu 2001), and peanuts (Hashim et al. 1993). However, the interactive effects of temperature and drought on fatty acid composition of vegetative organs require further studies, as these two factors often occur simultaneously and can exacerbate plant stress responses (Qaderi et al. 2010; Swann 2018). As shown, the interactive effects of increased temperature and drought are significantly more destructive than their individual effects (Prasad et al. 2011; Mahrookashani et al. 2017).

In this study, canola was used to examine the individual and interactive effects of temperature and watering regime on growth and physiological traits and determine essential fatty acids in the vegetative parts of this plant species. Canola is an important oilseed crop that contributes heavily to the Canadian economy, bringing in approximately $15.4 billion annually (Rempel, Hutton, and Jurke 2014). Fatty acids act as regulators of stress signaling in plants (He and Ding 2020). We hypothesized that higher temperature and drought stress adversely affect canola growth and its fatty acids by altering physiological processes. The objectives of this study were (i) to determine the individual and interactive effects of temperature and watering regime on canola growth and physiological traits, and (ii) to explore the effects of these factors on fatty acids of vegetative organs (leaf and stem) in this species.

## 2 Materials and Methods

### 2.1 Plant and Growth Conditions

The seeds of canola ( *Brassica napus* L. cv. 6056 CR, BrettYoung Seeds, Winnipeg, MB, Canada) were used to study the effects of temperature and drought on plant growth and development. Three replications of 50 seeds were germinated on one layer of blue filter paper (Anchor Paper Co, St. Paul, MN, USA) in 10 × 1.5 cm Petri dishes. The filter paper was moistened with 10 mL distilled water before placing the seeds in the Petri dishes. The Petri dishes were placed in a growth chamber (model ATC26; Conviron, Winnipeg, MB, Canada), which was set to a temperature regime of 22°C for 16 h day and 18°C for 8 h night, with a photosynthetic photon flux density (PPFD) of 300 μmol photons m^−2^ s^−1^. The seeds were regularly watered and separated if the root systems became too large. Following 5 days of germination, the seedlings were transferred to pots containing a mixture of peat moss, perlite, and vermiculite (2:1:1 ratio by volume) and watered to field capacity. An estimated 35 pellets of slow‐release fertilizer (N‐P‐K, 14‐14‐13 with micronutrients; Chisso‐Asahi Fertilizer Co, Tokyo, Japan) were added to each pot. The seedlings were placed in the same low‐temperature chamber for 7 days to allow the seedlings to acclimate to the environment. The emergence of true leaves indicates that the plants were ready to be put under experimental conditions. The seedlings were randomly assigned to one of the four experimental conditions: (1) lower temperatures (22°C/18°C), well‐watered (watering to field capacity, determined by drainage of excess water); (2) lower temperatures, water stressed (watering at wilting point); (3) higher temperatures (28°C/24°C), well‐watered; and (4) higher temperatures, water stressed. The lower‐temperature regime is the normal growth temperature for canola plants and the higher‐temperature regime simulates an increase in air temperature by the end of this century, as mentioned above. In both chambers, with lower and higher temperatures, the light was supplied by a mix of cool white, fluorescent lamps (Sylvania Pentron FP39/841/HO/ECO, Redding, CA, USA), and incandescent bulbs (Lumens 40 W/120 V; Sonepar, Laval, QC, Canada) on a 16‐h photoperiod. The trays were rotated twice a week, and the individual pots were rotated every other day to minimize any variations within the chambers. In each of the four treatments (with 10 plants), we measured plant growth and physiological traits. Experiments were conducted three times, and each set of measurements contained three replications per trial.

### 2.2 Measurement of Leaf and Soil Water Potential and Leaf Moisture

The water potential of leaf (from three fully grown leaves) and soil for each treatment were taken using the Dew Point PotentioMeter (WP4C Dew Point PotentiaMeter; Decagon Devices, Pullman, WA, USA). Before measurement, the device was calibrated with potassium chloride (AquaLab; Hoskin Scientific Ltd., Burlington, ON, Canada). The leaves were detached from the plant and placed at the bottom of the plastic caps. A thin layer of soil was acquired to cover the bottom of a metal cap. Covers were added to reduce moisture loss from the leaves and soil. Water potential was expressed in MPa (Abo Gamar et al. 2019).

Leaf moisture was measured for each treatment by first weighing the fresh mass (FM) of leaves with an analytical balance and then drying them at 60°C in the oven for 24 h and reweighing to obtain dry mass (DM) of leaves, as following: moisture % = (leaf FM − leaf DM)/leaf FM × 100 (Qaderi et al. 2012).

### 2.3 Measurement of Gas Exchange

Net carbon dioxide assimilation (*A*_N_, μmol CO_2_ m^−2^ s^−1^), transpiration (*E*, mmol H_2_O m^−2^ s^−1^), and stomatal conductance (*g*_s_, mol H_2_O m^−2^ s^−1^) were determined for three plants from each treatment using a portable photosynthesis system (Model LI‐6400XT; LI‐COR Biosciences, Lincoln, NE, USA). From each plant, two measurements were taken. Instantaneous WUE (μmol CO_2_ mmol H_2_O^−1^) was calculated by dividing *A*_N_ by *E*. The infrared gas analyzer (IRGA) was calibrated to a CO_2_ concentration of 400 μmol mol^−1^. The light emitted from the LED sources was calibrated to 300 μmol photon m^−2^ s^−1^, which was the light intensity used in this study (Martel and Qaderi 2016).

### 2.4 Measurement of Chlorophyll Fluorescence

From each treatment, three fully grown leaves were randomly selected to measure chlorophyll fluorescence. A portable fluorometer (Fluorpen FP 100; Photon Systems Instruments, Drasov, Czech Republic) was used to measure chlorophyll fluorescence. The effective quantum yield of photosystem II (ϕPSII) was determined in the light‐adapted leaves by measuring photosynthetic electron transport. After that, the leaves were dark adapted within the fluorometer clamp for 30 min to measure maximum quantum yield of PSII (*F*_v_/*F*_m_), nonphotochemical quenching (qNP), and photochemical quenching (qP; Baker 2008). A saturating pulse of 2100 μmol photons m^−2^ s^−1^ was delivered for 1 s (Johnson et al. 2009; Martel and Qaderi 2021).

### 2.5 Measurement of Photosynthetic Pigments

From each treatment, three fully grown leaves were used to measure the concentrations of chlorophyll (Chl) *a*, Chl *b*, and carotenoids. From each leaf, two discs were taken with a hole puncher and placed into a 12‐mL vial containing 2.5 mL of dimethyl sulfoxide (VWR International Inc., West Chester, PA, USA). The samples were incubated at room temperature in the dark for 24 h. The absorbances were read using a UV/visible spectrophotometer (Model Ultraspec 3100 *pro*; Biochrom Ltd., Cambridge, UK) and measured at 664, 648, and 470 nm (Chappelle, Kim, and McMurtrey III 1992). Then, the concentrations of Chl *a*, Chl *b*, carotenoids, total Chl, and Chl *a*:*b* ratio were calculated (Qaderi, Kurepin, and Reid 2006).

### 2.6 Measurement of NBI, Flavonoids, and Anthocyanin

Nitrogen balance index (NBI), flavonoids, and anthocyanin were determined with a Dualex Scientific (Dualex Scientific, Force‐A, OrsayCedex, France), which measures optical absorbance. NBI shows the estimated plant nitrogen status; it is based on the ratio of chlorophyll and flavonoids. Dualex determines flavonoids through quantitative comparison of light delivered to and emitted from the leaf. The relative absorbance units for flavonoids and anthocyanin (0 to 3 and 0 to 1.5, respectively) are reported in μg cm^−2^ (Martel and Qaderi 2016).

### 2.7 Measurement of Plant Growth and Biomass

From each treatment, three plants, which were grown under the experimental conditions for 3 weeks, were harvested to measure biomass, which included leaf mass, stem mass, root mass, and total mass. Stem height, leaf area, and leaf number were also measured. Stem height was measured with a ruler and stem diameter with a Digimatic caliper (Mitutoyo Corp., Kanagawa, Japan) before placing plants under experimental conditions and at the conclusion of the experiment. Plant mass was measured by drying the plant parts for 72 h at 60°C in a forced‐air Fisher Isotemp Premium Oven (model 750F; Fisher Scientific, Nepean, ON, Canada). The aboveground and belowground biomass were weighed on an analytical balance (model ED244s; Sartorius, Goettingen, Germany). The leaf area was measured with an area meter (Delta‐T Devices, Cambridge, UK). From these measurements, growth indices were calculated. The growth indices, used in this study, were SLM (leaf DM/leaf area, g m^−2^), LMR (leaf DM/plant DM), LAR (leaf area/plant DM, cm^2^ g^−1^), and shoot–root mass ratio (SRR) (shoot DM/root DM; Qaderi, Kurepin, and Reid 2012).

### 2.8 Measurement of Fatty Acids

At the conclusion of the experiment, from each treatment three plants that were grown for 3 weeks under the experimental conditions were randomly selected and their leaves and stems were cut with scissors and wrapped tightly in tinfoil. Plant samples were placed at −80°C for at least 3 days. The stems and leaves were then freeze dried for up to 120 h using a freeze dryer (BenchTop Pro, Omnitronics; SP Scientific, Gardiner, NY, USA). They were then stored at −20°C until chemical analysis was performed. The dried leaves and stems were ground into a powder using a mortar and pestle. After grinding, 50 mg of the powder was weighed using an analytical balance (model ED224s; Sartorius, Goettingen, Germany) and transferred to a 5‐mL centrifuge tube. To this powder, 0.19 mL of a mixture of chloroform, methanol, and distilled water (1:2:0.8, v/v/v) was added. The samples were then incubated at 4°C for 30 min at 117 rpm (Innova 4000; New Brunswick Scientific, Edison, NJ, USA). Then, 0.05 mL of 1.5% sodium sulphate was added to each vial and centrifuged (Heraeus Fresco 17; Thermo Scientific, Waltham, MA, USA) for 15 min. The upper polar layer was extracted using a pipette and transferred to a new 2‐mL vial. The lower nonpolar layer was discarded. Then, 0.05 mL internal standard solution was added to the new vials (Salimon, Omar, and Salih 2014). The internal standard solution was prepared by dissolving 18 mg of pentadecanoic acid (C15:0) in 9 mL of methanol. The samples were then dried using a vacuum concentrator (Concentrator Plus/Vacufuge Plus; Eppendorf, Hamburg, Germany).

The dried samples were redissolved in 0.2 mL *n*‐hexane. Then, 0.1 mL of 2 M methanolic potassium hydroxide (KOH) was added and vortexed for 30 s. The samples were then placed in a water bath at 70°C for 2 min. After incubation, 0.12 mL of 1.0 M hydrochloric acid (HCl) was added to each vial and stirred to initiate phase separation. Once this occurred, 0.1 mL of *n*‐hexane was added and gently stirred. The upper layer, which contained FAMEs (fatty acid methyl esters), was transferred into GC vials (Salimon, Omar, and Salih 2014).

To quantify the fatty acids, present in the leaves and stems of canola, standard solutions of linoleic acid, alpha‐linolenic acid, and gamma‐linolenic acid were prepared. Various concentrations of the standards were prepared to determine the concentration of fatty acids. A 1 μL standard solution of fatty acids was injected by an autosampler (model G4513A; Agilent, Santa Clara, CA, USA) into a gas chromatograph–flame ionization detector system (Model 7820A‐G4350B; Agilent) stocked with a capillary column (DB‐23, 30 m × 0.32 mm × 0.25 μm; Agilent). In the GC‐FID, the temperature of the injector and detector was set to 250°C, and the carrier gas of helium was set to 30 mL min^−1^. A 1 μL of each plant sample was injected into the GC‐FID with a split ratio of 10:1. After the injection of sample, the oven temperature was held at 175°C for 3 min, then increased to 230°C at a rate of 4°C min^−1^, and this temperature was held for 10 min (David, Sandra, and Vickers 2005). Based on this information, we can compare the fatty acids present in plant samples to the standard fatty acids. A standard FAME solution (Supelco Inc., Bellefonte, PA, USA) was used to calculate the amounts of fatty acids in the samples (Zou and Wu 2023). Fatty acids are reported as ng μL^−1^ of plant sample.

### 2.9 Data Analysis

The effects of temperature, drought, and their interactions were determined on growth and physiological traits of canola, using analysis of variance (ANOVA) for split‐plot design (SAS Institute 2016). In this analysis, temperature was the main plot factor, watering regime the subplot factor, and trial (chamber) as replication. The means were compared among main‐plot levels (temperature) within a common subplot level (watering regime), and subplot levels (watering regime) within a common main‐plot level (temperature), for traits with significant interaction using least significant differences (LSD) at the 5% confidence level (SAS Institute 2016). For fatty acids, the effects of temperature, watering regime, and plant organ were determined by ANOVA for split‐split‐plot design (SAS Institute 2016). In the split‐split‐plot analysis, temperature was considered as the main plot, watering regime as the subplot, plant organ as the split subplot, and growth chamber as replication (Hinkelmann and Kempthorne 2008). Differences among treatments were determined by one‐way ANOVA using LSD at the 5% confidence level (SAS Institute 2016). The relationship between plant traits was determined by treatment means by Pearson's correlation coefficient (Minitab Inc. 2021). All data are reported as mean ± SEM (standard error of the mean).

## 3 Results

### 3.1 Plant Growth

Overall, the stem height of canola plants was significantly affected by temperature and watering regime, but not by their interaction (Table 1). Plants grown under higher temperatures were relatively taller than those grown under lower temperatures (Table 2). The well‐watered plants were significantly taller compared to the water‐stressed plants (Table 2).

**TABLE 1:** Summary of split‐plot ANOVA (*F* value) for effects of temperature and watering regime on various traits of canola ( *Brassica napus* ) plants.

Source	df	Plant growth				Dry mass			
		Stem height	Stem diameter	Leaf area	Leaf number	Leaf	Stem	Root	Total
Temperature (*T*)	1	270.40**	23.30*	3.46	1.60	1.25	24.66*	1.39	3.15
Main plot error	2	—	—	—	—	—	—	—	—
Watering regime (*W*)	1	11.46**	23.68***	23.58***	6.10*	7.59*	33.22***	4.04	26.12***
*T* × *W*	1	1.64	0.26	0.94	0.98	3.11	8.91*	2.14	10.42**
Subplot error	10	—	—	—	—	—	—	—	—

Source	df	Growth index				Gas exchange			
		SLM	LMR	LAR	SRR	*A*_N_	*E*	*g*_s_	WUE
Temperature (*T*)	1	8.09	0.35	3.74	30.76*	1.80	0.01	0.06	22.21*
Main plot error	2	—	—	—	—	—	—	—	—
Watering regime (*W*)	1	1.52	16.04**	0.34	0.68	6.48*	10.57**	11.30**	0.53
*T* × *W*	1	1.53	1.08	1.49	0.13	0.03	0.14	0.07	0.02
Subplot error	10	—	—	—	—	—	—	—	—

Source	df	Chlorophyll fluorescence				Photosynthetic pigments				
		ϕPSII	*F*_v_/*F*_m_	qNP	qP	Chl *a*	Chl *b*	Carotenoids	Total Chl	Chl *a:b*
Temperature (*T*)	1	26.98*	5.05	6.26	5.38	2.24	12.39	39.52*	3.93	23.36*
Main plot error	2	—	—	—	—	—	—	—	—	—
Watering regime (*W*)	1	2.44	3.13	0.06	1.31	2.28	0.09	0.00	1.38	2.08
*T* × *W*	1	1.66	1.91	1.25	0.08	15.25**	8.39*	12.48***	14.08**	0.43
Subplot error	10	—	—	—	—	—	—	—	—	—

Source	df	Growth indicator	Protective compounds		Water potential		Moisture
		NBI	Flavonoids	Anthocyanin	Leaf	Soil	Leaf
Temperature (*T*)	1	67.63*	10.18	9.31	31.77*	0.00	2.06
Main plot error	2	—	—	—	—	—	—
Watering regime (*W*)	1	0.34	0.02	0.74	0.10	2.57	4.92
*T* × *W*	1	0.01	0.09	0.74	0.35	3.96	0.07
Subplot error	10	—	—	—	—	—	—

*Note:* Plants were grown under two temperature regimes (22°C/18°C, lower; 28°C/24°C, higher; 16 h light/8 h dark) and two watering regimes (watering to field capacity, well‐watered; watering at wilting point, water stressed) in controlled‐environment growth chambers for 21 days, after 1 week of initial growth under control conditions.

Abbreviations: *A*_N_, net CO_2_ assimilation; Chl, chlorophyll; *E*, transpiration; *F*_v_/*F*_m_, maximum quantum yield of PSII; *g*_s_, stomatal conductance; LAR, leaf area ratio; LMR, leaf mass ratio; NBI, nitrogen balance index; qNP, nonphotochemical quenching; qP, photochemical quenching; SLM, specific leaf mass; SRR, shoot: root mass ratio; WUE, water use efficiency; ϕPSII, effective quantum yield of PSII.

* *p* < 0.05.

** *p* < 0.01.

*** *p* < 0.001.

**TABLE 2:** Effects of temperature and watering regime on plant growth, dry mass, growth index, gas exchange, chlorophyll fluorescence, photosynthetic pigment, water potential, leaf moisture, growth indicator, and protective compound of canola ( *Brassica napus* ) plants. Subsequent comparison between main plot (temperature) and subplot (watering regime) levels for plant traits with nonsignificant interactions in split‐plot analysis.

Trait	Temperature		Watering regime	
	Lower	Higher	Well‐watered	Water stressed
Plant growth				
Stem height (cm)	2.56 ± 0.39a	3.21 ± 0.67a	3.91 ± 0.52a	1.86 ± 0.26b
Stem diameter (mm)	4.69 ± 0.30a	3.43 ± 0.28b	4.68 ± 0.28a	3.44 ± 0.30b
Leaf area (cm^2^ plant^−1^)	11.37 ± 1.73a	14.48 ± 2.58a	17.63 ± 1.80a	8.22 ± 0.87b
Leaf number (plant^−1^)	7.62 ± 0.42a	8.12 ± 0.40a	8.50 ± 0.19a	7.25 ± 0.45b
Dry mass				
Leaf mass (mg)	503.81 ± 50.65a	564.15 ± 68.98a	628.15 ± 52.95a	439.81 ± 47.38b
Root mass (mg)	101.91 ± 14.55a	167.22 ± 79.13a	207.20 ± 71.66a	61.94 ± 11.64a
Growth index				
Specific leaf mass (g m^−2^)	4.98 ± 0.43a	3.66 ± 0.17b	4.06 ± 0.30a	4.57 ± 0.48a
Leaf mass ratio	0.69 ± 0.02a	0.64 ± 0.17a	0.61 ± 0.03b	0.71 ± 0.02a
Leaf area ratio (cm^2^ g^−1^)	14.44 ± 1.14a	17.72 ± 1.27a	15.58 ± 1.23a	16.58 ± 1.45a
Shoot:root mass ratio	1.46 ± 0.22a	1.93 ± 0.50a	1.95 ± 0.50a	1.44 ± 0.22a
Gas exchange				
*A*_N_ (μmol CO_2_ m^−2^ s^−1^)	6.75 ± 1.06a	5.70 ± 0.91a	7.83 ± 0.77a	4.62 ± 0.84b
*E* (mmol H_2_O m^−2^ s^−1^)	2.62 ± 0.45a	2.59 ± 0.59a	3.58 ± 0.49a	1.63 ± 0.20b
*g*_s_ (mol H_2_O m^−2^ s^−1^)	0.19 ± 0.03a	0.18 ± 0.06a	0.28 ± 0.05a	0.09 ± 0.01b
WUE (μmol CO_2_ mmol H_2_O^−1^)	3.41 ± 0.87a	2.41 ± 0.25a	2.53 ± 0.42a	3.29 ± 0.82a
Chlorophyll fluorescence				
ϕPSII	0.68 ± 0.05a	0.74 ± 0.01a	0.75 ± 0.00a	0.68 ± 0.05a
*F*_v_/*F*_m_	0.77 ± 0.04a	0.82 ± 0.01a	0.83 ± 0.00a	0.76 ± 0.03a
qNP	1.02 ± 0.10a	0.84 ± 0.09a	0.95 ± 0.10a	0.91 ± 0.10a
qP	0.22 ± 0.03a	0.19 ± 0.03a	0.23 ± 0.02a	0.18 ± 0.03a
Photosynthetic pigment				
Chlorophyll *a:b* ratio	3.35 ± 0.15a	3.14 ± 0.07a	3.37 ± 0.15a	3.13 ± 0.04a
Water potential				
Leaf water potential (MPa)	−0.80 ± 0.03a	−1.38 ± 0.07b	−1.08 ± 0.12a	−1.11 ± 0.13a
Soil water potential (MPa)	−0.42 ± 0.03a	−0.43 ± 0.04a	−0.39 ± 0.03a	−0.46 ± 0.04a
Leaf moisture (%)	89.21 ± 2.66a	84.47 ± 1.84a	90.04 ± 1.42a	83.64 ± 2.67a
Growth indicator				
Nitrogen balance index	32.82 ± 1.36b	45.01 ± 2.44a	37.99 ± 2.44a	39.85 ± 3.54a
Protective compound				
Flavonoids (μg cm^−2^)	1.06 ± 0.05a	0.91 ± 0.03b	0.99 ± 0.03a	0.98 ± 0.06a
Anthocyanin (μg cm^−2^)	0.22 ± 0.01a	0.24 ± 0.00a	0.23 ± 0.01a	0.23 ± 0.01a

*Note:* Plants were grown under two temperature regimes (22°C/18°C, lower; 28°C/24°C, higher; 16 h light/8 h dark) and two watering regimes (watering to field capacity, well‐watered; watering at wilting point, water stressed) in controlled‐environment growth chambers for 21 days, after 1 week of initial growth under control conditions. Data are means ± SEM from three replicated experiments. Means followed by different letters within rows for each trait and factor are significantly different (LSD, *p* < 0.05).

Abbreviations: *A*_N_, net CO_2_ assimilation; *E*, transpiration; *F*_v_/*F*_m_, maximum quantum yield of PSII; *g*_s_, stomatal conductance; qNP, nonphotochemical quenching; qP, photochemical quenching; WUE, water use efficiency; ϕPSII, effective quantum yield of PSII.

Temperature and watering regime significantly affected stem diameter (Table 1). Plants under lower temperatures had thicker stems compared to plants under higher temperatures (Table 2). The water‐stressed plants had thinner stems than the well‐watered plants (Table 2).

Leaf area was significantly affected by watering regime, but not by temperature (Table 1). Plants under lower temperatures had relatively smaller leaves than plants under higher temperatures (Table 2). The water‐stressed plants had significantly smaller leaves compared to the well‐watered plants (Table 2). Some water‐stressed leaves were discolored in both temperature regimes.

Leaf number was significantly affected by watering regime, but not by temperature or by the interaction of temperature and watering regime (Table 1). The well‐watered plants had significantly more leaves compared to the water‐stressed plants (Table 2).

### 3.2 Dry Mass

Leaf mass was significantly affected by watering regime, but not by temperature or by the interaction of temperature and watering regime (Table 1). The water‐stressed plants had reduced leaf mass compared to the well‐watered plants (Table 2).

Root mass was not significantly affected by temperature, watering regime, or the interaction of these factors (Table 1). Although not significant, the root mass was reduced in plants under lower temperatures and in the water‐stressed plants (Table 2). Upon observation, the root systems of water‐stressed plants appeared to be longer than the root systems of well‐watered plants.

In DM with significant interactions, stem mass was significantly affected by temperature and watering regime and the interaction of these factors (Table 1). The well‐watered plants under higher temperatures had significantly higher stem mass compared to the well‐watered plants under lower temperatures. The water‐stressed plants under higher temperatures had significantly lower stem mass than the well‐watered plants under the same temperature regime (Table 3). Overall, the well‐watered plants grown under higher temperatures had the highest stem mass, whereas the water‐stressed plants grown under lower temperatures had the lowest stem mass (Table 3).

**TABLE 3:** Effects of temperature and watering regime on stem and total dry mass, and photosynthetic pigments of canola ( *Brassica napus* ) plants. Subsequent comparison between main plot levels (temperature) within a common subplot level (watering regime), within rows; or between subplot levels (watering regime) within a common main plot level (temperature), within columns and traits; for plant traits with significant interactions in split‐plot analysis.

Trait	Watering regime	Temperature	
		Lower	Higher
Stem mass	Well‐watered	162.25 ± 20.84Ba	304.80 ± 33.32Aa
	Water stressed	96.03 ± 20.60Aa	96.33 ± 7.88Ab
Total mass	Well‐watered	821.60 ± 68.55Ba	1316.20 ± 122.79Aa
	Water stressed	648.10 ± 126.15Aa	547.70 ± 29.02Ab
Chlorophyll *a*	Well‐watered	6.84 ± 0.29Ba	8.68 ± 0.22Aa
	Water stressed	7.73 ± 0.31Aa	6.67 ± 0.53Ab
Chlorophyll *b*	Well‐watered	1.98 ± 0.20Ab	2.72 ± 0.16Aa
	Water stressed	2.44 ± 0.10Aa	2.17 ± 0.16Aa
Carotenoids	Well‐watered	1.12 ± 0.18Ba	1.84 ± 0.02Aa
	Water stressed	1.54 ± 0.05Aa	1.41 ± 0.12Ab
Total chlorophyll	Well‐watered	8.82 ± 0.45Ba	11.40 ± 0.75Aa
	Water stressed	10.17 ± 0.40Aa	8.83 ± 0.68Ab

*Note:* Plants were grown under two temperature regimes (22°C/18°C, lower; 28°C/24°C, higher; 16 h light/8 h dark) and two watering regimes (watering to field capacity, well‐watered; watering at wilting point, water stressed) in controlled‐environment growth chambers for 21 days, after 1 week of initial growth under control conditions. Data are means ± SEM from three replicated experiments. Means followed by different uppercase letters within rows or by different lowercase letters within columns and traits are significantly different (LSD, *p* < 0.05). Stem and total dry mass (mg); photosynthetic pigments (μg cm^−2^).

Also, total mass was significantly affected by the watering regime and the interaction of temperature and watering regime (Table 1). The well‐watered plants under higher temperatures had significantly higher total mass compared to the well‐watered plants under lower temperatures. The well‐watered plants under higher temperatures had significantly higher total mass than the water‐stressed plants under the same temperature regime (Table 3). Overall, the well‐watered plants under higher temperatures had the highest total mass, whereas the water‐stressed plants under the same temperature regime had the lowest total mass (Table 3).

### 3.3 Growth Index

Temperature, watering regime, and the interaction of these factors did not significantly affect SLM and LAR based on split‐plot analysis (Table 1). However, plants under lower temperatures had higher SLM than plants under higher temperatures based on one‐way ANOVA (LSD, *p* < 0.05; Table 2).

LMR was significantly affected only by the watering regime (Table 1). The well‐watered plants had significantly lower LMR compared to the water‐stressed plants (Table 2).

SRR was significantly affected by temperature, but not by watering regime or by the interaction of temperature and watering regime (Table 1). Plants that experienced lower temperatures or water stress had relatively decreased SRR than plants that experienced higher temperatures or watering to field capacity, respectively (Table 2).

### 3.4 Gas Exchange

Net CO_2_ assimilation (*A*_N_) was significantly affected by watering regime (Table 1). The well‐watered plants had significantly higher *A*_N_ compared to the water‐stressed plants (Table 2). Neither temperature nor the interaction of temperature and watering regime significantly affected *A*_N_ (Table 1).

Transpiration (*E*) was also significantly affected by watering regime (Table 1). The well‐watered plants had significantly higher *E* compared to the water‐stressed plants (Table 2). Temperature or the interaction of temperature and watering regime did not significantly affect *E* (Table 1).

The watering regime, but not temperature or interactions of these factors, had significant effects on stomatal conductance (*g*_s_; Table 1). The well‐watered plants had significantly higher *g*_s_ than the water‐stressed plants (Table 2).

Temperature, but not watering regime or the interaction of temperature and watering regime, significantly affected WUE (Table 1). Plants under lower temperatures had relatively increased WUE than plants under higher temperatures (Table 2).

### 3.5 Chlorophyll Fluorescence

Effective quantum yield of photosystem II (ϕPSII) was significantly affected by temperature, but not by watering regime or by the interaction of these factors (Table 1). Plants under higher temperatures had relatively increased ϕPSII than plants under lower temperatures (Table 2).

Neither maximum quantum yield (*F*_v_/*F*_m_) nor nonphotochemical quenching (qNP) and photochemical quenching (qP) were significantly affected by temperature, watering regime, and their interactions (Tables 1 and 2).

### 3.6 Photosynthetic Pigments

In all pigments, there were significant interactions between temperature and watering regime. Chlorophyll (Chl) *a*, Chl *b*, carotenoids, and total Chl were all significantly affected by the two‐way interaction of temperature and watering regime (Table 1). The well‐watered plants under higher temperatures had significantly higher Chl *a* than the well‐watered plants under lower temperatures (Table 3). The water‐stressed plants under higher temperatures had significantly lower Chl *a* than the well‐watered plants under the same temperature regime (Table 3). Overall, the well‐watered plants under higher temperatures had the highest Chl *a*, whereas the water‐stressed plants under the same temperature regime had the lowest Chl *a* (Table 3).

The water‐stressed plants under lower temperatures had higher Chl *b* compared to the well‐watered plants under the same temperature regime (Table 3). Overall, the well‐watered plants under higher temperatures had the highest Chl *b*, whereas the well‐watered plants under lower temperatures had the lowest Chl *b* (Table 3).

The well‐watered plants under higher temperatures had significantly higher carotenoids compared to the well‐watered plants under lower temperatures. The well‐watered plants under higher temperatures had significantly higher carotenoids than the water‐stressed plants of the same temperature regime (Table 3). Overall, the well‐watered plants under higher temperatures had the highest carotenoids, whereas the well‐watered plants under lower temperatures had the lowest carotenoids (Table 3).

The well‐watered plants under higher temperatures significantly affected total chlorophyll content. The well‐watered plants under higher temperatures had significantly higher total Chl compared to the water‐stressed plants of the same temperature regime. Overall, the well‐watered plants under higher temperatures had the highest total Chl, whereas the well‐watered plants under lower temperatures had the lowest total Chl (Table 3).

However, in Chl *a:b* ratio, there was no significant interaction between temperature and watering regime. Chl *a:b* ratio was significantly affected by temperature, but not by watering regime or by the interaction of temperature and watering regime (Table 1). Chl *a*:*b* ratio was relatively lower under higher temperatures than under lower temperatures (Table 2).

### 3.7 Leaf and Soil Water Potential and Leaf Moisture

Leaf water potential was significantly affected by temperature, but not by watering regime or by the interaction of these factors (Table 1). Leaf water potential was lower in plants under higher temperatures than those under lower temperatures (Table 2).

Soil water potential and leaf moisture were not significantly affected by temperature, watering regime, and the interaction of these factors (Tables 1 and 2).

### 3.8 NBI, Flavonoids, and Anthocyanin

NBI was significantly affected by temperature, but not by watering regime or by the interaction of the two factors (Table 1). Plants under higher temperatures had significantly higher NBI compared to plants under lower temperatures (Table 2).

Flavonoids and anthocyanin were not significantly affected by temperature, watering regime, and the interaction of these factors based on split‐plot analysis (Table 1). However, higher temperatures decreased flavonoids based on one‐way ANOVA (LSD, *p* < 0.05; Table 2).

### 3.9 Fatty Acids

In our study, both saturated and unsaturated fatty acids were detected in plant samples; from which palmitoleic acid (C16:1), heptadecanoic acid (C17:0), linoleic acid (C18:2 *cis*), and heneicosanoic acid (C21:0) were not significantly affected by temperature, watering regime, plant organ, and the interactions of these factors based on split‐split‐plot analysis (Table 4). In fatty acids with nonsignificant interactions, there was a significant difference in C16:1 between leaves and stems based on one‐way ANOVA (LSD, *p* < 0.05; Table 5). C16:1 was significantly higher in the stems than in the leaves (Table 5). C17:0 was increased, but tricosanoic acid (C23:0) was decreased, by higher temperatures (Table 5).

**TABLE 4:** Summary of split‐plot ANOVA (*F* value) for effects of temperature and watering regime on fatty acids of canola ( *Brassica napus* ) plants.

Source	df	Fatty acid				
		C16:1	C17:0	C17:1	C18:1 *trans* (n9)	C18:2 *cis*
Temperature (*T*)	1	2.94	0.14	21.92*	37.11*	3.90
Main plot error	2	—	—	—	—	—
Watering regime (*W*)	1	0.00	0.32	0.07	2.23	0.75
*T* × *W*	1	1.12	2.96	0.00	5.12	0.41
Subplot error	4	—	—	—	—	—
Organ (*O*)	1	1.65	1.62	3.92	0.54	1.46
*T* × *O*	1	0.94	1.23	0.56	0.71	0.01
*W* × *O*	1	0.11	1.84	5.54	1.73	0.11
*T* × *W* × *O*	1	0.00	1.67	11.19*	7.39*	4.11
Split‐plot error	8	—	—	—	—	—

Source	df	Fatty acid				
		C18:3 (n6)	C20:0	C20:2	C21:0	C23:0
Temperature (*T*)	1	54.87*	0.38	74.23*	0.08	0.35
Main plot error	2	—	—	—	—	—
Watering regime (*W*)	1	0.09	0.14	1.97	0.26	14.95*
*T* × *W*	1	0.10	0.46	0.00	0.71	0.41
Subplot error	4	—	—	—	—	—
Organ (*O*)	1	0.00	3.88	1.05	1.52	2.82
*T* × *O*	1	0.08	0.67	0.54	0.11	0.14
*W* × *O*	1	0.03	3.84	7.59*	1.00	1.00
*T* × *W* × *O*	1	1.28	13.09**	11.15*	0.49	3.74
Split‐plot error	8	—	—	—	—	—

*Note:* Plants were grown under two temperature regimes (22°C/18°C, lower; 28°C/24°C, higher; 16 h light/8 h dark) and two watering regimes (watering to field capacity, well‐watered; watering at wilting point, water stressed) in controlled‐environment growth chambers for 21 days, after 1 week of initial growth under control conditions. Fatty acids (ng μL^−1^).

Abbreviations: C16:1, palmitoleic acid; C17:0, heptadecanoic acid; C17:1, *cis*‐10‐heptadecenoic acid; C18:0, stearic acid; C18:1, *trans* (n9), elaidic acid; C18:2 *cis*, linoleic acid; C18:3 (n6), γ‐linolenic acid; C20:0, arachidic acid; C20:1 (n9), *cis*‐11‐eicosanoic acid; C20:2, *cis*11,14‐eicosadienoic acid; C21:0, heneicosanoic acid; C23:0, tricosanoic acid.

* *p* < 0.05.

** *p* < 0.01.

**TABLE 5:** Effects of temperature, watering regime, and plant organ on fatty acid content of canola ( *Brassica napus* ) plants. Subsequent comparison among main plot (temperature), subplot (watering regime), and split subplot (plant organ) levels for plant traits with nonsignificant interactions in split‐split‐plot analysis.

Trait	Temperature		Watering regime		Plant organ	
	Lower	Higher	Well‐watered	Water stressed	Leaf	Stem
C16:1	31.20 ± 1.84a	31.29 ± 1.46a	32.55 ± 1.58a	29.94 ± 5.00a	28.46 ± 1.46b	34.03 ± 1.32a
C17:0	25.5 ± 1.28b	35.68 ± 3.18a	29.17 ± 2.72a	29.818 ± 2.60a	28.11 ± 2.08a	31.15 ± 3.33a
C18:2 *cis*	10.73 ± 1.84a	11.23 ± 1.61a	12.11 ± 1.36a	9.46 ± 2.08a	11.66 ± 1.73a	10.25 ± 1.77a
C18:3 (n6)	12.33 ± 1.79a	14.14 ± 1.93a	13.86 ± 1.75a	12.44 ± 1.97a	14.48 ± 1.84a	11.56 ± 1.77a
C21:0	14.03 ± 2.20a	14.88 ± 3.11a	16.12 ± 3.06a	12.66 ± 2.15a	14.08 ± 2.81a	14.84 ± 2.63a
C23:0	11.75 ± 0.87a	6.71 ± 1.13b	8.02 ± 1.10a	10.81 ± 1.28a	9.84 ± 1.33a	8.90 ± 1.18a

*Note:* Plants were grown under two temperature regimes (22°C/18°C, lower; 28°C/24°C, higher; 16 h light/8 h dark) and two watering regimes (watering to field capacity, well‐watered; watering at wilting point, water stressed) in controlled‐environment growth chambers for 21 days, after 1 week of initial growth under control conditions. Data are means ± SEM from three replicated experiments. Means followed by different letters within rows for each trait and factor are significantly different (LSD, *p* < 0.05). Fatty acid (ng mL^−1^).

Abbreviations: C16:1, palmitoleic acid; C17:0, heptadecanoic acid; C18:2 *cis*, linoleic acid; C18:3 (n6), γ‐linolenic acid; C21:0, heneicosanoic acid; C23:0, tricosanoic acid.

In fatty acids with significant interactions, γ‐linolenic acid (C18:3; n6) was significantly affected by temperature, whereas the fatty acid C23:0 by watering regime based on split‐split‐plot analysis (Table 4). The fatty acids *cis*‐10‐heptadecenoic acid (C17:1) and elaidic acid (C18:1) were significantly affected by temperature and the three‐way interactions of *T* (temperature) × *W* (watering regime) × *O* (plant organ), arachidic acid (C20:0) by the three‐way interaction, and *cis*‐11,14‐eicosadienoic acid (C20:2) by temperature, the two‐way interaction of *W* × *O*, and the three‐way interaction (Table 4).

The leaves of the well‐watered plants under higher temperatures had significantly higher C17:1 than the leaves of the well‐watered plants under lower temperatures and the leaves of the water‐stressed plants under higher temperatures (Table 6). In the water‐stressed plants under lower temperatures as well as in the well‐watered plants under higher temperatures, C17:1 was higher in the leaves than in the stems (Table 6).

**TABLE 6:** Effects of temperature, watering regime, and plant organ on fatty acid content of canola ( *Brassica napus* ) plants. Subsequent comparison among main plot (temperature) and subplot (watering regime) levels within a common split‐subplot level (plant organ), within rows; or between split‐subplot (plant organ) levels within main plot (temperature) and subplot (watering regime), within columns and traits; for plant traits with significant interactions in split‐split‐plot analysis.

Trait	Plant organ	Lower temperature		Higher temperature	
		Well‐watered	Water stressed	Well‐watered	Water stressed
C17:1	Leaf	41.40 ± 4.57BCa	51.80 ± 8.38ABa	62.77 ± 1.86Aa	33.28 ± 1.26Ca
	Stem	36.11 ± 7.50Aa	28.32 ± 3.17Ab	33.64 ± 4.39Ab	32.80 ± 4.21Aa
C18:1 *trans* (n9)	Leaf	67.09 ± 6.70ABa	85.0 ± 17.14 Aa	81.05 ± 5.71Aa	44.23 ± 5.40Ba
	Stem	26.63 ± 1.39Ab	15.01 ± 4.23Ab	18.22 ± 7.07Ab	21.66 ± 6.59Aa
C20:0	Leaf	206.36 ± 24.40ABa	255.41 ± 41.57Aa	272.68 ± 10.49Aa	125.34 ± 11.43Ba
	Stem	224.27 ± 46.33Aa	174.82 ± 16.63Aa	204.21 ± 30.96Aa	213.16 ± 31.90Aa
C20:2	Leaf	412.66 ± 30.47Ba	591.77 ± 123.15Aa	608.60 ± 3.37Aa	284.08 ± 43.60Ba
	Stem	267.98 ± 54.67Aa	231.88 ± 20.50Ab	183.15 ± 24.59Ab	195.29 ± 21.53Aa

*Note:* Plants were grown under two temperature regimes (22°C/18°C, lower; 28°C/24°C, higher; 16 h light/8 h dark) and two watering regimes (watering to field capacity, well‐watered; watering at wilting point, water stressed) in controlled‐environment growth chambers for 21 days, after 1 week of initial growth under control conditions. Data are means ± SEM from three replicated experiments. Means followed by different uppercase letters within rows or by different lowercase letters within columns and traits are significantly different (LSD, *p* < 0.05). Fatty acid (ng mL^−1^).

Abbreviations: C17:1, *cis*‐10‐heptadecenoic acid; C18:1 *trans* (n9), elaidic acid; C20:0, arachidic acid; C20:2, *cis*11,14‐eicosadienoic acid.

The leaves of the water‐stressed plants under higher temperatures had significantly lower C18:1 than the leaves of the water‐stressed plants under lower temperatures and the leaves of the well‐watered plants under higher temperatures (Table 6). Except for the water‐stressed plants under higher temperatures, the leaves of other treatments had significantly higher C18:1 than the stems (Table 6).

Like C18:1, the leaves of the water‐stressed plants under higher temperatures had significantly lower C20:0 than the leaves of the water‐stressed plants under lower temperatures and the leaves of the well‐watered plants under higher temperatures (Table 6).

In the leaves, the water‐stressed plants under lower temperatures and the well‐watered plants under higher temperatures had significantly increased C20:2, whereas the well‐watered plants under lower temperatures and the water‐stressed plants under higher temperatures had significantly decreased C20:2 (Table 6). In the water‐stressed plants under lower temperatures and the well‐watered plants under higher temperatures, C20:2 was higher in the leaves than in the stems (Table 6).

It is also important to note that some other fatty acids were present in trace amounts in our samples. These fatty acids include lauric acid (C12:0), stearic acid (C18:0), oleic acid (C18:1), α‐linolenic acid (C18:3), *cis*‐8,11,14‐eicosatrinoic acid (C20:3), and *cis*‐10‐eicosanoic acid (C20:1; n9), which are not reported here due to insufficient concentration in some of the replications.

## 4 Discussion

In this study, we explored the individual and interactive effects of two components of climate change, temperature and watering regime, on the vegetative growth of canola—an oilseed crop in Canada and around the world. Overall, temperature, watering regime, and their interactions significantly influenced many traits of plants, including growth, physiological properties, and fatty acids (Tables 1 and 4).

### 4.1 Effects of Temperature

In this study, we found that canola plants grown under higher temperatures had relatively taller and significantly thinner stems than those grown under lower temperatures (Table 2). Since increased temperature can accelerate plant metabolism, it results in thinner stems and larger root systems to counteract water loss that is associated with higher temperatures (Qaderi, Kurepin, and Reid 2006). The relatively larger LAR under higher temperatures shows that this temperature regime leads to larger leaves (Rawson, Gardner, and Long 1987; Craufurd et al. 1999). Rapid cell elongation under higher temperatures is associated with a larger number of cells per leaf unit area, supporting our findings (Rosbakh, Römermann, and Poschlod 2015). The relatively larger leaf area under higher temperatures increased photosynthetic rates, contributing to the overall increase in total DM (Lambers and Oliveira 2019). Reduced SLM under higher temperatures indicates that this temperature regime causes the leaves to be thinner than those developed under lower temperature regime (Table 2). A lower SLM under higher temperatures is a method to induce leaf cooling (Qaderi, Godin, and Reid 2015).

In this study, decreased leaf water potential under higher temperatures indicates decreased evapotranspiration rates in these plants. This may be caused by a decrease in the pressure gradient between leaves and atmosphere, which drives the hydrologic cycle (Craufurd et al. 1999). Increased NBI under higher temperatures shows that plants under this condition have higher total chlorophyll concentration than flavonoids; decreased flavonoid content under this temperature regime indicates this outcome (Table 2). Also, decreased flavonoid content indicates that plants under higher temperatures are less protected because of their role as antioxidant compounds, protecting plants against ROS (Bita and Gerats 2013).

In this study, higher temperatures relatively increased the concentrations of some fatty acids with nonsignificant interaction (Table 5). In fatty acids with significant interaction, higher temperatures increased them in the well‐watered plants but decreased in the water‐stressed plants (Table 6). It has been shown that environmental stressors, such as higher temperatures, can influence fatty acids, most notably the ratio of oleic acid to linoleic acid can change when plants experience high temperatures at maturity (Omidi et al. 2010). Linolenic acid has been shown to decrease under higher temperatures (Schulte et al. 2013). In this study, oleic acid was detected in a very low concentration in the stems and leaves. Although, in our study, arachidic acid was not significantly affected by temperature, an earlier study revealed that higher temperatures increased the concentration of this fatty acid in soybean (Rennie and Tanner 1989).

### 4.2 Effects of Watering Regime

In this study, water stress significantly decreased all aspects of plant growth (Table 2). Decreased leaf area resulted in reduced net CO_2_ assimilation (*A*_N_), transpiration (*E*), and stomatal conductance (*g*_s_), but increased WUE. It has been shown that WUE is high when the stomatal conductance is low in comparison to the assimilated CO_2_ (Lambers and Oliveira 2019). This indicates that water stress was effective in reducing other gas exchange traits while simultaneously increasing WUE. It has also been found that decreased *g*_s_ has a dominant role in the downregulation of *A*_N_ in water‐stressed plants (Bota, Medrano, and Flexas 2004). A previous study has shown that water stress combined with higher temperatures reduced *A*_N_, resulting in decreased plant DM (Qaderi, Kurepin, and Reid 2012). In our study, the root mass was not significantly affected by water stress (Table 2), which is unexpected as one of the hallmarks of water stress in plants is the extension of root systems (Barnabás, Jäger, and Fehér 2008; Qaderi, Kurepin, and Reid 2012; Fang and Xiong 2015). In theory, there should have been a significant difference in root mass between the well‐watered and water‐stressed plants.

In this study, watering regime did not have significant effects on leaf and soil water potential, and leaf moisture (Table 2). A previous study has shown that leaf and soil water potential and leaf moisture decreased under water stress in Arabidopsis ( *Arabidopsis thaliana* ; Abo Gamar et al. 2019). Reduced moisture by water stress was also found in canola (Qaderi et al. 2010). In addition, NBI, flavonoids, and anthocyanin were not significantly affected by watering regime (Table 2), indicating that this cultivar is less sensitive to water stress. Flavonoids and anthocyanin are common indicators of plant stress (Fang and Xiong 2015) and the results revealed that less sensitivity of this cultivar to water stress allows plants to modify their growth characteristics to cope with environmental stressors.

In this study, an increase in heptadecanoic acid (C17:0) and a decrease in tricosanoic acid (C23:0) by higher temperatures (Table 5) indicate that heat stress likely increases short‐chain fatty acids but decreases long‐chain fatty acids. In canola oil, C17:0 is found in small amounts; therefore, most studies neglect reporting this fatty acid in their samples (Ratnayake 2015). In our study, water stress did not have such an effect on fatty acids (Table 5). An earlier study has shown that, in canola, water stress decreases oleic acid (C18:1), linoleic acid (C18:2), and behenic acid (C22:0), but increases palmitic acid (C16:0), stearic acid (C18:0), arachidic acid (C20:0), and linolenic acid (C18:3; Dawood and Sadak 2014). However, this was not the case in our study. In this study, C16:1 is the only fatty acid that is higher in the stem than in the leaf, suggesting that the concentrations of most fatty acids are similar between the plant organs. An earlier study, in the oil of canola greens, has shown that the percentage of unsaturated fatty acids is higher than that of saturated fatty acids, and the percentage of polyunsaturated fatty acids is higher than that of monounsaturated fatty acids (Bhardwaj, Hamama, and Rangappa 2003); however, in our study, such clear patterns were not found.

### 4.3 Interactive Effects of Temperature and Watering Regime

In this study, higher temperatures increased stem and total mass in the well‐watered plants, whereas water stress decreased them under higher temperatures only (Table 3). It seems that the effects of higher temperatures are pronounced in the well‐watered plants when comparing the two temperature regimes, and the effects of water stress are within the higher temperature regime. In well‐watered plants, heat stress causes the stomata to open to cool (Gommers 2020); however, when heat stress is combined with water stress, stomatal opening is adversely affected (Qaderi, Kurepin, and Reid 2006). Reduced uptake of CO_2_, due to partial closure of stomata, leads to the overall reduction of *A*_N_, resulting in decreased plant biomass (Barnabás, Jäger, and Fehér 2008), as seen in our study. In water‐stressed plants under higher temperatures, the source–sink relations may change; it has been noted that the interactive effects of heat and water stress are more severe than the individual effects (Mahrookashani et al. 2017).

In this study, the well‐watered plants under higher temperatures had increased photosynthetic pigments, but the water‐stressed plants had decreased photosynthetic pigments under the same temperature regime (Table 3). Also, the pattern of photosynthetic pigments, including Chl *a*, carotenoids, and total Chl, is like that of plant biomass (Tables 2 and 3). In this study, such similarity indicates the role of photosynthetic pigments in the biomass production of plants under critical temperature and water.

In our study, higher temperatures in the well‐watered plants increased *cis*‐10‐heptadecenoic acid and *cis*‐11,14‐eicosadienoic acid (Tables 5 and 6). In an earlier study, it was shown that moderately high temperature increased the oil:protein ratio of all three cultivars of canola, which were considered heat tolerant, heat intermediate, and heat sensitive; however, it was stated that minor fatty acids, such as those mentioned here, did not show consistent trends (Aksouh‐Harradj, Campbell, and Mailer 2006). Decreased C20:0 in the leaf of water‐stressed plants under higher temperatures (Table 6) indicates the synergistic effects of temperature and water stress on this fatty acid, which deserves further studies.

## 5 Conclusions

In this study, plant growth and gas exchange were negatively influenced by the individual effects of water stress, whereas the effects of higher temperature were not as drastic. In terms of fatty acids, the combined effects of temperature, watering regime, and plant organ were more pronounced than the individual effects of these factors. Overall, higher temperatures combined with water stress decreased some physiological traits and fatty acids and, in turn, plant biomass. A combination of higher temperatures, water stress, and leaf (as plant organ) generally decreased fatty acids. Also, the stems of water‐stressed plants under lower temperatures and the stems of well‐watered plants under higher temperatures had decreased fatty acids. This indicates that climate change can influence plant fatty acids, which differ between/among plant organs. Further studies are required to determine the effects of multiple climate change components on fatty acid composition and its regulatory role in stress response in canola and other oilseed crops.

## Conflicts of Interest

The authors declare no conflicts of interest.

## Data Availability

All the required data are provided in the manuscript.
